# Effect of Self-reported Physical Activity on Glycaemia and Blood Pressure in Healthy Participants from Bissau: A Cross-sectional Study

**DOI:** 10.1186/s40798-025-00821-0

**Published:** 2025-02-25

**Authors:** Lilica Sanca, Cipriano Có, Nelson Namara, Aladje Lopes, Albino Emanuel, Bárbara Oliveiros, Stine Byberg, Morten Bjerregaard-Andersen, Eugénia Carvalho, Alain Massart, Ana Teixeira

**Affiliations:** 1 CNC UC - Center for Neuroscience and Cell Biology, University of Coimbra, Coimbra, Portugal; 2 CIDAF-Research Center for Sport and Physical Activity, Faculty of Sport Sciences, University of Coimbra, Coimbra, Portugal; 3 National Institute of Health, National Public Health Laboratory, Bissau, Guinea-Bissau; 4 Bandim Health Project, Av. Combatentes da Liberdade da Pátria (Hospital 3 de Agosto), Bairro D’Ajuda– Bissau, República da Guiné-Bissau Apartado 861: Bissau, Bissau, 1004 Guinea-Bissau; 5 Exercise and Health, Universidade Técnica de Lisboa, Faculdade de Motricidade Humana (UTL-FMH), Lisboa, Portugal; 6 Gymnasium Academy Có & Có SARL, Bissau, Guinea-Bissau; 7 National Diabetes Association (ANDD) and National Diabetes Clinic (CND), Bissau, Guinea-Bissau; 8 Steno Diabetes Center Copenhagen (SDCC), Copenhagen, Denmark; 9 Department of Endocrinology and Nephrology, University Hospital of South Denmark, Esbjerg, Denmark; 10 Steno Diabetes Center Odense (SDCO), Odense, Denmark; 11 Institute of Interdisciplinary Research, University of Coimbra, Coimbra, Portugal; 12 CIBB - Center for Innovative Biomedicine and Biotechnology, Faculty of Medicine, University of Coimbra, Coimbra, Portugal; 13 LBIM-Laboratory of Biostatistics and Medical Informatics, Faculty of Medicine, University of Coimbra, Coimbra, Portugal

**Keywords:** Physical activity, IPAQ, Fasting glycaemia, Non-communicable disease, Body mass index, Low-income countries

## Abstract

**Background:**

Recent data show a dramatic increase in non-communicable diseases in developing countries, including cardiovascular disease, obesity and diabetes. Most of these diseases may be preventable and to some extent treatable by alterations in physical activity. We investigated the correlations of physical activity levels, according to energy expenditure [measured in metabolic equivalent minutes per week (METs x min/week)] with fasting glycaemia levels, blood pressure, body compositions and anthropometric variables in participants from Bissau, Guinea-Bissau.

**Method:**

The International Physical Activity Questionnaire was used to assess levels of physical activity, by calculating METs x min/week. These were subsequently divided into low, moderate or high physical activity categories in a healthy group of participants. Fasting glucose, arterial blood pressure, body mass index and body fat were also assessed. Data was analyzed using the SPSS statistical software.

**Results:**

429 participants were included from sports and health facilities around the country. Of these, 187 were highly active (> 3000 MET × min/ week or at least 1500 METs × min/week) and had a mean age of 26.8 ± 7.5 years. 76% (327/429) were male and 24% were female (102/429). The mean energy expenditure was 4866.7 ± 1241.2 METs × min/week and the mean fasting glucose was 94.0 ± 14.1 mg/dl. 4.1% (18/429) of the participants had diabetes and 12.8% (55/429) had hypertension. A significant negative correlation was found between blood glucose and total physical activity (r_s_ = − 0.117, *p* = 0.015). Sedentary participants had the highest proportion (5.1%) of diabetes and higher rates of hypertension (15.8%). These participants had the highest obesity (10.1%) and overweight prevalence (23.7%). In addition, they also presented the highest percentage (5.1%) of body fat, with 55% of them showing high or very high visceral fat% content. In the sedentary group, a significant positive correlation, was found, indicating a low moderate association, between systolic blood pressure and weekly sitting time, r_s_(175) = 0.205, *p* = 0.006.

**Conclusion:**

While high levels of physical activity had a significant impact on glycaemia control, physical activity had no significant impact on blood pressure. However, weekly sitting time may increase blood pressure.

## Key Points

• Higher levels of physical activity were associated with better glycemic control, lower body and visceral fat percentage, highlighting the protective role of physical activity against cardio metabolic risks.

• Sedentary individuals exhibited significantly higher visceral fat percentages, emphasizing its potential link to impaired glucose metabolism and hypertension.

• Structured physical activities, such as sports, demonstrated stronger correlations with blood glucose regulation compared to other domains of physical activity.

## Introduction

The World Health Organization (WHO) classifies diabetes mellitus, cardiovascular disease and obesity as non-communicable diseases (NCDs) that cause major threats to health, economies and societies [1, 2]. NCDs are collectively responsible for 74% of all deaths worldwide [3]. More than three-quarters of all NCD deaths occur in low- and middle-income countries [3]. NCDs usually develop over long time periods. These share four major risk factors impacted by poor health behaviors: tobacco use, physical inactivity, harmful use of alcohol and unhealthy nutrition. Personal factors such as age, ethnicity, and genetic factors are not amenable to change [3].

Most NCDs may be preventable by decreasing metabolic and behavioral risk factors [2]. There is evidence that physical activity (PA) may prevent various health conditions [4–6], including overweight and obesity [2, 7, 8], type 2 diabetes mellitus [1, 2, 9], hypertension [2, 10, 11] and hyperglycemia [2, 12].

PA has been regarded as one of the most important behaviors leading to a healthy life by preventing diseases and increasing health benefits [5, 13]. Attempts have been made to develop appropriate measurement tools, including objective and subjective measurement tools, to quantify the amount of daily PA [5]. Of these, questionnaires remain the most widely used in large-scale studies, due to their efficiency and ease of measuring PA levels in large populations (14). The International Physical Activity Questionnaire (IPAQ) is an instrument developed by the WHO designed to assess the levels of regular PA from the young to the elderly [5].

In Guinea-Bissau, the IPAQ for PA assessment has never been used, and there is a lack of specific scientific evidence on the effects of physical activity on glycaemia and blood pressure in healthy participants from this population. This study aimed to investigate the levels of physical activity, according to energy expenditure [measured in metabolic equivalent minutes per week (METs x min/week)], their correlation with fasting glycaemia levels, blood pressure, as well as anthropometric and body composition variables related to health, in participants from Bissau, Guinea-Bissau. The time estimates of energy expenditure used in the IPAQ assume that individuals can accurately determine the intensity and duration of their PA [14]. This study will contribute to the literature on the benefits of PA interventions in combating cardiometabolic diseases, particularly in Africa, where evidence on this topic is currently very limited.

## Method

### Recruitment and Sample Size

Participants aged 18 to 57 years were recruited between January 30 and February 16, 2023. Recruitment initially took place in the capital at different sport facilities, targeting physically active individuals engaged in athletic activities, physical exercise, sports training, or specific sports such as basketball, handball, and volleyball. These participants were regular users of sport facilities, including students from the National School of Physical Education and Sport or national athletes.

Additionally, a sedentary group of young to middle-aged adults was recruited from a larger cohort of 3000 individuals participating in a nationwide diabetes prevalence study conducted across the country. All included sedentary participants, who reported no engagement in sports activities in the questionnaire, were matched with physically active participants. To ensure the reliability of the study results, the sedentary and active groups were matched based on various risk factors, including sex, age, body mass, stature, family history of diabetes, diabetes diagnosis, tobacco use, and alcohol consumption. This matching process aimed to minimize the influence of these factors on the analysis and interpretation of the results.

Similar studies using the IPAQ questionnaire have achieved significant results with both small and large sample sizes [15]. For this study, a larger sample size was considered important to ensure adequate statistical power and a high probability of detecting true differences between categories, if present. However, practical considerations, such as the availability of resources and the time required for recruitment and data collection, limited the number of participants. Additionally, the pool of subjects meeting the necessary characteristics for the study was restricted. As a result, the final sample size was determined to be feasible within these constraints.

### Inclusion Criteria

General Population: male or female participants, young to middle-aged, residing in Guinea-Bissau, who were not undergoing treatment for/or having a history of cardiovascular diseases, cancer, insulin-dependent diabetes, neurodegenerative diseases, or other significant medical conditions. Participants must have provided written informed consent and completed all required data for the study.

Physically Active Group: participants who were national athletes or students of physical education and sports, engaging in a minimum of three training sessions per week.

Sedentary Group: Participants with no regular sports practice. To ensure comparability with the active group, the sedentary group included individuals with certain characteristics such as non-insulin-dependent diabetes, tobacco use, and alcohol consumption.

### Data Collection

During recruitment, participants completed a questionnaire to provide personal information and were informed about the procedures and preparations required for the data collection day. On the collection day, early in the morning, under 8 to 12 h fasting conditions and at rest, the blood pressure and the glucose levels were measured. In addition, anthropometric and body composition measurements were done, and the IPAQ-Long form questionnaire [16] to evaluate the Physical Activity level of participants was filled in.

#### Anthropometric Measures

Measures were performed according to the International Society for Advancement of Kinanthropometry (ISAK) recommendations [17].

Body stature was measured using a steel ting measuring tape of 3 meters, push-down blade lock (HENGNUO brand, 16’ model, China), for accurate measurement.

Body mass (BM) was measured using a bioimpedance scale Omron Karada Scan BF511 (blue model, Omron brand, Germany). Body mass index (BMI) was calculated using mass and stature according to the equation BMI = Body mass kg/stature × m^2^.

Waist (WC), hip (HC) and mid-upper arm circumference (MUAC), were recorded using a flexible tape measure PRIMA 150 cm (Mètre ruban model, Meetlint brand, France). The waist to hip ratio (WHR) range was classified according to WHO definition: as low (men, ≤ 0.95 and women, ≤ 0.80), moderate (men, ≤ 0.95 < 1.0 and women, ≤ 0.80 < 0.85) and high (men, ≥ 1.0 and women, ≥ 0.85) [18]. Waist to stature ratio (WSR) was also estimated.

#### Body Composition Measures

A body composition monitor with Bioelectrical Impedance, Omron Karada Scan BF511 (blue model, Omron brand, Germany) characteristics, was used according to the guidelines from the manufacturer, to measure the percentage of body fat (BF%), visceral fat (VF%) and skeletal muscle (SM%). A previous study, in Guinea-Bissau, comparing the performance of two bioimpedance scales, showed a good agreement between the Omron Karada Scan BF511 and the Tanita^®^ BC-545 [18].

The cut-offs for body composition were those defined by the manufacturer of the Omron Karada Scan BF51 analyzer. The Omron report provided include weight (kg), BF ((%), VF (%), BMI. (kg/m^2^). The principal cut-off points as recommended by WHO, were used to categorize the BMI: underweight (< 18.5 kg/m^2^), normal weight (18.5 to < 25 kg/m^2^), overweight (25 to < 30 kg/m^2^) or obese (≥ 30 kg/m^2^) [19]. For body fat percentage the cut-off is High (BF ≥ 23.0 < 40.0), Very High (BF ≥ 40.0) and for visceral fat percentage the cut-off isHigh (VF ≥ 10.0 < 15.0) and Very High (VF ≥ 15.0).

#### Glycaemia Measures

Fasting glycaemia levels were measured using a glucometer from Roche, the AccuCheck Instant (Blood Glucose meter AccuCheck Instant model, Roche brand, Switzerland). A finger blood drop was taken from all fasting participants. Participants were classified as having pre-diabetes if their fasting glucose levels ranged from 100 to 125 mg/dL [according to American Diabetes Association (ADA) guidelines]. They were classified as having diabetes if their fasting glucose levels were > 126 mg/dL or if they reported a prior diabetes diagnosis [12].

#### Blood Pressure Measures

Blood pressure was measured using a Wellion WAVE professional Blood Pressure Monitor (Wellion model, WellionVet brand, Austria) using the oscillometric method. The participants were asked to stay relaxed and try to remain still for 5–10 min before taking a measurement. Subjects were measured twice, with a 1- to 2-minute interval between measurements, on the left arm at the same height than the heart, sitting on a chair with backrest, staying calm and avoiding talking, laughing or coughing [20]. Hypertension, was considered when values of Systolic Blood Pressure (SBP) and Diastolic Blood Pressure (DBP) were > 140/90 mm Hg (SBP > 140 / DBP > 90) after two repeated measures were recorded, or if the participants were already being treated with anti-hypertensive agents [21]. Ethnicity (local ethnic group), current smoking, and drinking habits were also reported.

#### IPAQ Measures

One-week total PA was assessed using the Portuguese version of the long-form International Physical Activity Questionnaire (IPAQ) [22]. All the study participants were literate and had access to a watch for time estimation according to the domains of IPAQ. For each self-reported PA category, metabolic equivalents (MET) of energy expenditure were estimated [22]. These were calculated as a total of occupation section, transportation section, housework section, and sport/recreational activity section reported in MET × min/week [22]. The current guidelines for PA in the IPAQ scoring protocol categorizes PA into three groups of low, moderate or high levels [23]. Total PA was categorized as low (< 600 MET × min/week), moderate (600–3000 MET × min/week), and high (> 3000 MET × min/week or a moderate total PA including at least 1500 METs × min/week with vigorous activity in minimum 3 days of the week) [20–24]. Only activities that lasted at least 10 min were considered [16, 22].

### Statistical Analysis

Statistical analysis was performed using the IBM^®^ SPSS^®^ Statistics 29.0 package (SPSS Inc., Chicago, USA). A descriptive analysis of the data was performed given means, standard deviations, and percentage. Normality of continuous variables was checked by the Shapiro-Wilk test [25] to determine the use of parametric tests (t test, ANOVA, Pearson) [26] or non-parametric tests (Mann and Whitney, Kruskal-Wallis, Spearman) [27]. For both ANOVA and Kruskal-Wallis tests, post-hoc comparison using the Pairwise method with a Bonferroni correction for multiple tests was used [28]. The Chi-square test was performed to determine whether there were significant (*p* = 0.05) differences between the categorical variables. Linear regression was used to find a model that best explained glycaemia, systolic blood pressure, or diastolic blood pressure as dependent variables [29]. Only significant coefficient independent variables were selected, and this explained the higher percentage of the variance in the dependent variable, and the assumptions of linear regression were checked. Size effect of the obtained statistics was used to check how meaningful the results were [30].

## Results

### Group Characteristics

From the 436 participants enrolled, 429 completed all the information requested (questionnaires, tests) and the 7 with missing data were excluded from the analysis.

The assessment of the METs× min/week allowed for the separation of participants into three groups, regarding physical activity levels. One group with moderate Physical Activity (MPA) (*n* = 65, 15%), one group of high level of Physical Activity (HPA) (*n* = 187, 44%) and one sedentary behavior (SB) group of (*n* = 177, 41%).

As shown in Table 1, the total METs × min/week was respectively 618.7 ± 75.4, 2220.0 ± 592.2 and 4766.1 ± 1247.0 for SB, MPA and HPA groups respectively. Kruskal-Wallis test with post-hoc comparisons using with Bonferroni corrections, indicated that the sedentary group presented significantly higher sitting time per week, and lower energy spent in transportation, housework, and sport/recreational PA domains than the MPA and the HPA groups. The HPA group showed significantly higher PA in all domains compared to the MPA group.

**Table 1:** Physical activities in METs.minutes per week and Time sitting per week in minute among the groups

IPAQ domain	Sedentarily (Mean ± SD) *N* = 177	Moderate level Physical Activity (Mean ± SD) *N* = 65	High level Physical Activity (Mean ± SD) *N* = 187	H, *P* value
Occupational	235.82 ± 108.4	201.26 ± 342.6	1163.70 ± 1001.5	*P* < 0.001
Transport	159.50 ± 91.4	454.73 ± 324	769.62 ± 518.9	*P* < 0.001
Yard/garden/Household	223.43 ± 94.2	440.12 ± 436.8	1065.18 ± 702.2	*p* < 0.001
Sport/Leisure	0 ± 0	1123.89 ± 456.8	1764.60 ± 889.9	*p* < 0.001
Total IPAQ	618.75 ± 75.5	2220.05 ± 592.2	4763.20 ± 1247	*P* < 0.001
Sit time by week	3667.48 ± 1029.1	1895.50 ± 1192.7	2000.76 ± 1455.8	*p* < 0.001

*H* = Kruskal-Wallis tests

The distribution of sex, stature, body mass, family history of diabetes, previous diagnosis of diabetes and tobacco was not statistically different between the groups, but fasting glycaemia, age and alcohol consumption were higher in the SB and MPA group compared with the HPA group (see Table 2).

**Table 2:** Characteristics of study participant among the three groups

	Sedentary (*n* = 177)	Moderate level Physical Activity (*n* = 65)	High level Physical Activity (*n* = 187)	
Variables	Mean ± SD or % (*n*/*N*)	Mean ± SD or % (*n*/*N*)	Mean ± SD or % (*n*/*N*)	*P* value
*n*/*N* (%)	41 (177/429)	15 (65/429)	44 (187/429)	
Gender (M, %)	76 (134/177)	75 (49/65)	77 (144/187)	*X*^2^, *p* = 0.994
Age (Years)	31 ± 10.5	32 ± 10.7	28 ± 8.4	*H*, *p* = 0.012
Stature (m)	1.68 ± 0.09	1.68 ± 0.08	1.70 ± 0.08	*F*, *p* = 0.217
Weight (kg)	68.6 ± 16.4	63.8 ± 10.1	64.5 ± 9.8	*H*, *p* =0 0.118
BMI (kg/m²)	24.0 ± 5.2	22.4 ± 3	22.2 ± 3.1	*H*, *p* = 0.024
Body Fat (%)	25.7 ± 11.2	19.7 ± 7.5	18.7 ± 8.4	*H*, *p* <0.001
Visceral Fat (%)	6.0 ± 4.2	4.2 ± 4.1	3.3 ± 2.7	*H*, *p* <0.001
Skeletal Muscle (%)	34.1 ± 7.4	37.3 ± 4.1	37.8 ± 4.7	*H*, *p* <0.001
Waist (cm)	80.6 ± 14.1	76.2 ± 15.6	74.5 ± 9.4	*H*, *p* < 0.001
Hip (cm)	95.7 ± 11.6	91.8 ± 7.7	91.6 ± 9.1	*H*, *p* = 0.002
Waist/Hip	0.84 ± 0.1	0.83 ± 0.2	0.81 ± 0.1	*H*, *p* = 0.001
Waist/Stature	0.47 ± 0.1	0.45 ± 0.1	0.43 ± 0.1	*H*, *p* < 0.001
MUAC (cm)	29.9 ± 6.4	28.2 ± 3	27.7 ± 3.4	*H*, *p* < 0.001
Fasting Glycaemia (mg/dl)	98.9 ± 31.9	95.6 ± 30	92.9 ± 14	*H*, *p* = 0.044
Diagnose of Diabetes (%)	5 (9/177)	5 (3/65)	2 (4/187)	*X*^2^, *p* = 0.307
Systolic Pressure (mm Hg)	130.8 ± 25.4	127.9 ± 14.7	129.7 ± 19.6	*H*, *p* = 0.882
Diastolic Pressure (mm Hg)	80.0 ± 15.2	80.7 ± 10.8	81.8 ± 13.2	*H*, *p* = 0.579
Tobacco Consumption (%)	9 (15/177)	8 (5/65)	4 (7/187)	*X*^2^, *p* = 0.157
Alcohol consumption (%)	21 (37/177)	39 (25/65)	22 (41/187)	*X*^2^, *p* = 0.012
Diabetes Family history (%)	16 (28/177)	14 (9/65)	17 (31/187)	*X*^2^, *p* = 0.257

*H* = Kruskal-Wallis tests, *X*^2^ = Chi-square test, BMI = body mass index, MUAC = mid upper arm circumference

### Anthropometric and Body Composition Results

Except for body mass and stature, as shown in Table 2, the Kruskal-Wallis test indicated significant differences between the three groups in all the anthropometric and body composition measured. Post-hoc comparison using the Pairwise method with a Bonferroni correction for multiple tests, indicated a significant health status difference, in favor of the HPA group compared to the SB group, for BMI, WC and HC, WHR and WSR. In addition, it indicated a significant health status difference in favor of HPA and MPA group compared to the SB group for SM%, BF% and VF%. MUAC measurement showed significant differences, with the SB group presenting higher values than the HPA group.

### Blood Pressure and Fasting Blood Glucose

In Table 2, the Kruskal-Wallis test did not indicate significant differences for SBP and DBP between groups, but it showed a significant difference in fasting glycaemia (*H*(*2*) = 6.24, *p* = 0.044). Post hoc pairwise comparisons using Bonferroni correction indicated significant (*p* = 0.02) fasting glycaemia difference, in favor of the HPA group compared to the SB group. The SB group had a mean fasting glycaemia of 98.9 mg/dl (95% CI: 94.91, 104.43) with a standard deviation of 31.9, and the HPA group had a mean fasting glycaemia of 92.9 mg/dl (95% CI: 91.12, 95.04) and a standard deviation of 14.

### Health risk Prevalence between Groups

According to the data presented in Table 3, SB exhibited the highest proportions of elevated fasting blood glucose levels, hypertension, and excess weight (30.1%, 15.8%, and 33.7%, respectively) compared to MPA and HPA, who showed similar results between them. The proportion of individuals with excess BF% was higher in the PA groups (∼ 25%) compared to the SB group (17.5%). However, VF excess was significantly more common in the SB group (56.5%) than in either of the PA groups (3–6%). Additionally, SB individuals had the highest percentage (5.1%) of diabetes, and presented high rates of hypertension similar to those of the HPA group (∼ 27%).

**Table 3:** Health risk percentage among the three groups

Health Status	Sedentary % (*n*/*N*)	Moderate level Physical Activity % (*n*/*N*)	High level Physical Activity % (*n*/*N*)
**Diabetes**			
Diabetes (F-glucose > 125 mg/dl)	5.1 (9/177)	4.6 (3/65)	3.2 (6/187)
Pre-Diabetes (F-glucose ≥ 100 ≤ 125 mg/dl)	25 (45/177)	14 (9/65)	19 (35/187)
**Blood Pressure**			
Hypertensive (SBP > 140| DBP > 90	15.8 (28/177)	9.2 (6/65)	11.2 (21/187)
**Obesity**			
Obese (BMI ≥ 30.0)	10.1 (18/177)	3.1 (2/65)	1.6 (3/187)
Overweight (BMI ≥ 25.0 < 30.0)	23.7 (42/177)	13.8 (9/65)	17.6 (33/187)
Underweight (BMI < 18.5 )	6.8 (12/177)	4.6 (3/65)	5.3 (10/187)
**Body composition**			
High (BF% ≥ 23.0 < 40.0)	12.4 (22/177)	27.7 (18/65)	22.9 (43/187)
Very High (BF% ≥ 40.0)	5.1 (9/177)	0.0 (0/65)	2.1 (4/187)
High (VF% ≥ 10.0 < 15.0)	41.8 (74/177)	3.1 (2/65)	2.6 (5/187)
Very High (VF% ≥ 15.0)	14.7 (26/177)	3.1 (2/65)	0.5 (1/187)

F = fasting, SBP = systolic blood pressure, DBP = diastolic blood pressure, BMI = body mass index, BF%=body fat percentage, VF%=visceral fat percentage

### Relationship between PA, Blood Glucose, Blood Pressure and Body Metrics

In Table 4, the correlations between IPAQ results and glycaemia or blood pressure, showed a significant negative correlation, albeit weak, between blood glucose and total IPAQ or Sport/leisure IPAQ (rs = − 0.117, *p* = 0.015), but no other associations were found.

**Table 4:** Correlation between the PA by IPAQ domains and blood glucose and blood pressure

Spearman’s rho	Total METS in IPAQ		Siting Time by week IPAQ		Sport IPAQ		Moderate PA		Vigorous PA	
Variables	*r*	*p* value	*r*	*p* value	*r*	*p* value	*r*	*p* value	*r*	*p* value
Glycemia	-0.117	0.015	0.043	0.379	-0.175	< 0.001	-0.077	0.113	-0.05	0.304
Systolic Blood Pressure	0.022	0.646	0.030	0.540	-0.0035	0.465	0.045	0.353	0.035	0.476
Diastolic Blood Pressure	0.026	0.597	-0.030	0.541	0.026	0.595	0.065	0.178	0.066	0.172

Moreover, significant positive correlations were found between MPA and fasting blood glucose (rs(427) = 0.241, *p* = < 0.001), BM (rs(427) = 0.302, *p* = < 0.001), BMI (rs(427) = 0.226, *p* = < 0.001), VF% (rs(427) = 0.242, *p* = < 0.001), WC (rs(427) = 0.251, *p* = < 0.001), MUAC (rs(427) = 0.265, *p* = < 0.001), and WHR (rs(427) = 0.241, *p* = < 0.001). The correlation analysis also suggested age and WC as potential variables impacting the fasting blood glucose.

Furthermore, linear regression (F(3,413) = 39.64, *p* = < 0.001) indicated that body mass, diabetes diagnosis, and energy expenditure in sports were the variables that most influenced fasting blood glucose (R 0.225), in a sample of the Guinean population consisting of sedentary individuals and athletes. MUAC, WC, VF% and BMI also exhibited significant positive correlations with fasting glucose level.

In addition, linear regression of the whole study population (F(2,412) = 56.85, *p* = < 0.001) indicated that body mass (R.112) and sex (R.031) were the variables most prone to positively influence SBP. Most body composition and anthropometric values (BM, BMI, VF%, WC, MUAC) showed a moderate positive correlation with SBP and DBP, while SM% and total IPAQ have a weak negative correlation with diastolic blood pressure. Only in the sedentary group, did weekly sitting time, show a significant positive correlation, with a low to moderate association, to both diastolic and systolic blood pressure. Linear regression indicates that BM and sex positively influence diastolic blood pressure, while SM% has a negative influence.

## Discussion

### Main Findings

Regarding PA, the SB group had significantly higher weekly sitting time and expended less energy on transportation, housework, and sports/recreational activities compared to the MPA and HPA groups.

No significant differences were found in blood pressure between the groups. However, body mass and sex together explained respectively 11.2 and 3.1% of systolic blood pressure variance across the entire population. Additionally, a weak negative association was observed between total IPAQ score and DBP.

In the SB group, weekly sitting time showed a positive association with both SBPand DBP, while muscles% was negatively associated with DBP.

Fasting blood glucose levels were significantly lower in the HPA group compared to the SB group and were negatively correlated with total and sport/leisure IPAQ scores. Positive, significant correlations were found between glucose levels and anthropometric or body composition values. BM, diabetes diagnosis, and sport/leisure IPAQ, together explained most of the variance in glucose levels.

Except for MUAC, all measured anthropometric and body composition values were more favorable in the HPA group compared to the SB group. When comparing the MPA group with the SB group, only body composition values showed a clear advantage. The SB group exhibited a higher prevalence of risk factors, with approximately 30% of subjects showing both excess BM and elevated fasting glucose levels, and 56.5% having high VF%.

### Physical Activity Levels

This study aimed to investigate the levels of PA (METs ×min/week), and the cross-sectional relationship between PA, fasting glycaemia and blood pressure, in different physically active groups, from sport and health facilities in Guinea-Bissau. This is the first study using the IPAQ in this country.

The WHO recommends a minimum of 1200 METs ×min/week as a benefit from healthy muscle activity, good blood circulation, reduction of glycaemia and obesity, to reduce the risk of NCD development [3]. The participants in the SB group exhibited values below the WHO recommendations, while the MPA and HPA groups demonstrated values that exceeded them, suggesting expected differences in health indicators between these groups.

Like in the study of Ojiambo et al. [31], in 97 Kenyan adolescent subjects, the most physically active subjects in the total IPAQ were also more active in all the domains (occupation, sport/leisure, transport and gardening) and spent less time sitting. In Africa, certain tasks in domestic work often require more than moderate physical activity, as more time is spent walking, due to lack of economic means to pay for public transportation or even the unavailability of public transports. The total IPAQ scores of our MPA and HPA groups are compatible with the general population results in other African adults of both sexes [32–34].

### Body Compositions and Anthropometric Variables

Despite having BM and stature measures equivalent to SB individuals, the HPA group had a significantly reduced BMI, BF%, VF%, as well as WC, HC, WHR, WSR, MUAC, and significantly higher levels of SM%. These results indicate that HPA levels are beneficial, as compared to SB individuals, regarding all health indicators measured in the study. The group with MPA had significantly lower levels of BF% and VF% compared to the SB group. These results highlight the significant impact of PA on anthropometric and body composition health indicators, with greater benefits as PA levels is increasing. Heydenreich et al., 2021 showed a good effect of PA on anthropometric and body composition variables, after 4 years follow-up in a group of university students [35], the contribution of PA to these variables being acknowledged [36, 37].

Interestingly, despite having mean BMI, WHR, and VF% values within the normal range and a lower BF% risk compared to the PA groups, the SB group had 56.5% subjects with VF% excess, compared to 6.2% and 3.1% in the MPA and HPA groups, respectively. This finding aligns with evidence suggesting that VF%, rather than BMI, may be a more sensitive indicator of cardio metabolic risk [38]. Elevated VF% has been closely linked to increased risks of type 2 diabetes and hypertension, likely due to its association with insulin resistance, chronic inflammation, and lipid metabolism disturbances [39]. These results highlight the importance of routine assessments of body composition and suggest that even individuals with a ‘healthy’ BMI should be encouraged to engage in regular physical activity to mitigate VF accumulation and associated risks.

While BM showed the highest correlation with fasting glucose and blood pressure and was the only common explanatory independent variable in the linear regression results, MUAC, WC, BMI, and VF% also exhibited high correlations, indicating the potential value of those variables in the prevention of cardiometabolic diseases in the study population.

### Blood Glucose

Compared to the SB group, the HPA group presented significantly lower blood glucose levels, indicating a protective effect of PA on glycaemia. A similar effect was not observed for those in the MPA group. As Miles, 2007 pointed out, in his study on PA and health, PA should cover a wide range of intensity and volume in order to minimize the development of insulin resistance, the duration of PA being an important factor [40]. Therefore, prescribing exercise that incorporates approximately 170 min of exercise per week substantially improves insulin sensitivity compared to programs that use only 115 min per week [40]. There is a trend towards decreasing blood glucose with increasing PA, but this association, although significant, is of little relevance for the MPA group. Nevertheless, increased energy expenditure in sports is likely to contribute to a decrease in blood glucose. Our results are in accordance with the American Diabetes Association [41] that report the benefit of exercise in decreasing blood glucose in subjects with type 2 diabetes and in preventing or delaying type 2 diabetes development, in reducing cardiovascular risk factors and in increasing weight loss.

### Blood Pressure

Schweiger et al. 2021 [10] indicate that even though elevated blood pressure levels are less common in the active population, athletes are not protected from hypertension. The prevalence of hypertension may vary by sport. Moreover, it appears to be even higher in athletes competing in certain disciplines compared to the general population [10]. We did not find differences in SBP or DBP between the groups of active subjects or SB individuals, nor did we obtain significant correlations between SBP or DBP and levels of PA.

While PA in the present study, was likely to modulate blood glucose, it did not have a significant impact on blood pressure. The weekly time spent sitting was positively associated with elevated SBP and DBP, reinforcing that physical inactivity could influence blood pressure more than physical activity. However, Bhammar et al., 2017 [42] found lower blood glucose and blood pressure after 30 min of continuous moderate-intensity walking in 10 participants. Nevertheless, a significant negative influence of the percentage of muscle mass on DBP, which was also evidenced with a weak association (r(325) = − 0.168, *p* = 0.002) to blood glucose, indicated that higher levels of muscle mass could be beneficial for the control of DBP and blood glucose. Development of hypertension is directly associated with BM and obesity, exercise has been long documented to improve blood pressure, even in obese individuals in absence of weight loss and one of the potential mechanisms is the increase in SM, which is modulated by myostatin, a fundamental negative regulator of muscle growth [43]. Results indicate that SM is a major site of insulin resistance in essential hypertension.

In agreement with Atkinson et al. [44], our study found that the male gender was associated with higher SBP and DBP and that diabetes diagnosis was significantly associated with increased blood glucose levels. While this second association is expected, the inclusion of both variables provides valuable insights, as a diabetes diagnosis reflects a clinical history, whereas blood glucose levels indicate current physiological status, capturing potential cases of undiagnosed diabetes or differences in disease control.

Sitting time was significantly higher in the SB group compared to the MPA and HPA groups. While SB behavior encompasses various low-energy activities, sitting time allows us to identify nuanced associations, as demonstrated by the significant relationship between sitting time and DBP in SB individuals. These findings align with previous research showing that prolonged sitting time is associated with an increased risk of hypertension and overall mortality [45, 46].

### Relationships among Key Study Variables in our Population

Body mass was the anthropometric variable that presented the most evident relationship with blood glucose and blood pressure. Other body composition and anthropometric variables such as BMI, VF%, WC, and MUAC were identified as good predictors of blood glucose and blood pressure. MUAC, a body composition measure frequently used as an indicator of malnutrition [47], was significantly higher in the SB group compared to the HPA group and presented a positive association with blood glucose and blood pressure. Those results may indicate a decrease in MUAC as PA increases, while the opposite was true for the SM%. A possible explanation is that MUAC measured in ourSB group included more fat around the arm relating to less PA. As stated by Li et al., 2022 skinfold thickness may be a convenient and credible indicator for the prediction of mortality, especially in those with severe cardiovascular diseases [48]. However, skinfold thickness was not measured in the present study, thus, we cannot verify this hypothesis as confirmed by Joglekar et al., 2007 [49] in 698 children at 6 years of age, for body proportions and growth from birth assessment. Zhu et al., 2020 showed that the MUAC was correlated with BMI, WC, WHR [50]. A greater MUAC was found by Hou et al., 2019 to be positively associated with higher risks of several cardio metabolic disorders as hypertension, subclinical atherosclerosis and diabetes in Chinese adults [51]. Similar results were found also by Zhu et al., 2020 where they defended that the MUAC is a simple and effective tool for the determination of central obesity and insulin resistance. Additionally, the larger the MUAC measures the more positive association with metabolic risk factors [50]. In another study carried by Wang et al., 2022 the WC and the MUAC were also negatively associated with insulin resistance in Chinese adults. And the association between MUAC and insulin resistance was derived from arm muscle instead of subcutaneous fat. The MUAC could be an additional predictor of insulin resistance in clinical practice, besides BMI and WC [52]. Additionally, our study found a moderate positive association between blood glucose and SBP (r(427) = 0.253, *p* = < 0.001), indicating that subjects with higher fasting blood glucose levels may also have higher blood pressure, confirming the findings of Quesada et al., 2021 [53].

### Study Limitations

One limitation of the study is that the sample is not representative of the entire population, as participants were recruited from a limited number of sports or health facilities. This may have introduced selection bias, as individuals using these facilities might be more health-conscious or physically active than the general population. For the SB group, while efforts were made to include controls, the recruitment process might still reflect a similar bias, as sedentary individuals visiting health facilities may differ in lifestyle or health awareness compared to the broader sedentary population.

We tried to match the groups and distribution of sex, stature, body mass, family history of diabetes, diagnosis of diabetes and tobacco consumption that were not statistically different between the groups. However, age and alcohol consumption which were elevated in the SB and the MPA group compared with the HPA group may have influenced the results of the study.

We did not evaluate the diet of the subjects, and potential confounders of glycaemia or blood pressure, such as the consumption of water, salt, potassium, carbohydrates, and alcohol, were not reported in detail or controlled for.

The PA assessment was subjective, as accelerometers were not available to objectively measure daily PA. Additionally, the accuracy of the portable bio-impedance and glycaemia tools used may be lower compared to laboratory instruments. In addition, hypertension and fasting glucose measurements were not repeated between two separate days to confirm the disease status.

## Conclusion

Compared to the SB group, the PA groups showed no significant differences in SBP and DBP. However, fasting blood glucose levels were significantly lower in the HPA group.

Positive and significant correlations were found between glucose levels and anthropometric or body composition variables, while negative correlations were observed with sport/leisure IPAQ scores. BM, diabetes diagnosis, and sport/leisure IPAQ together explained the majority of the variance in glucose levels.

The MPA and HPA groups exhibited significantly lower BF% and VF% and higher SM% compared to the SB group. Increased sitting time and a low SM% were associated with higher blood pressure in the SB group.

Compared to the SB group, the PA groups demonstrated significantly healthier BMI, WC and HC, WHR and WSR. MUAC measurements also differed significantly, with the SB group presenting higher values than the HPA group.

BM, BMI, VF%, WC, and MUAC were the body composition and anthropometric variables most strongly associated with blood glucose and blood pressure levels, suggesting their potential as predictors for the prevention of non-communicable diseases.

These findings underscore the critical role of PA in reducing cardiometabolic risks in Guinea-Bissau and across Africa, where evidence on this topic remains scarce. The high prevalence of VF excess, less favorable body composition and anthropometric outcomes, and elevated fasting blood glucose levels among SB individuals highlight the urgent need for targeted interventions to reduce sedentary behavior and promote structured physical activity, particularly vigorous activities that have a pronounced impact on VF reduction and metabolic health.

## Data Availability

The data can be made available by contacting the corresponding author upon reasonable request, provided approval by the study principal investigator (Ana Teixeira) and the Bandim Health Project scientific steering committee.

## Abbreviations

MET: Metabolic equivalent
IPAQ: International Physical Activity Questionnaire
WHO: World Health Organization
NCD: Non Communicable Disease
PA: Physical Activity
CNES: National Ethic Committee in Health studies
INASA: National Institute of Health
ISAK: International Society for Advancement of Kinanthropometry
BM: Body Mass
BMI: Body Mass Index
WC: Waist Circumference
HC: Hip Circumference
MUAC: Mid-Upper Arm Circumference
WHR: Waist to Hip Ratio
WSR: Waist to Stature Ratio
BF: Body fat
VF: Visceral Fat
SM: Skeletal Muscle
SBP: Systolic Blood Pressure
DBP: Diastolic Blood Pressure
